# Determinants of Low Birth Weight among Newborns Delivered at Public Hospitals in Sidama Zone, South Ethiopia: Unmatched Case-Control Study

**DOI:** 10.1155/2020/4675701

**Published:** 2020-04-16

**Authors:** Muse Bututa Bekela, Mulugeta Shegaze Shimbre, Teshale Fikadu Gebabo, Mengesha Boko Geta, Abayneh Tunje Tonga, Eshetu Andarge Zeleke, Negussie Boti Sidemo, Agegnehu Bante Getnet

**Affiliations:** ^1^Bona District Hospital, Bona, Sidama Zone, Ethiopia; ^2^College of Medicine and Health Sciences, School of Public Health, Arba Minch University, Ethiopia; ^3^College of Medicine and Health Sciences, School of Nursing, Arba Minch University, Ethiopia

## Abstract

Low birth weight is a global public health problem having various severe and life-threatening health effects. The World Health Organization is working to reduce the prevalence of low birth weight to 30% by the year 2025. Pinpointing the determinants of low birth weight at different scenarios is crucial to reduce the rate of low birth weight in low-income countries which consist of 96.5% of global low birth weight newborns. Thus, the aim of this study was to assess determinants of low birth weight in Sidama Zone public hospitals of South Ethiopia. An institution-based case-control study was conducted from March 1 to May 5, 2019, in Sidama Zone public hospitals. Data were collected from 354 mother-neonate samples with 118 of them having newborns with birth weight < 2500 g (cases) and 236 of them having birth weight ≥ 2500 g (controls) using a pretested, interviewer-administered structured questionnaire and medical record review. The odds of being rural dweller women was 3.51 times higher among cases (low birth weight babies) than among controls (normal birth weight babies) as compared to being urban dweller women (AOR = 3.51, 95% CI (1.91-6.45)). The likelihood of initiating antenatal care late was 3.22 times more among cases than among controls when compared with timely initiation of antenatal care (AOR = 3.22, 95% CI (1.47-7.14)). The probability of having pregnancy-induced hypertension was 4.49 times higher among mothers of the cases than among mothers of the controls as compared to not having pregnancy-induced hypertension (AOR = 4.49, 95% CI (1.94-10.38)). The odds of not taking iron and folic acid during pregnancy was 3.92 times higher among mothers of the cases than mothers of the controls when compared with taking iron and folic acid (AOR = 3.92, 95% CI (1.80-8.50)). The likelihood of having Mid-Upper Arm Circumference (MUAC) < 23 cm was 4.27 times higher among mothers of the cases than among mothers of the controls as compared to having MUAC ≥ 23 cm (AOR = 4.27, 95% CI (2.24-8.12)). The probability of having inadequate dietary diversity was 3.75 times higher among cases than among controls as compared to having adequate dietary diversity (AOR = 3.75, 95% CI (1.64-8.57)). Interventions targeting the aversion of low birth weight should focus on promotion of iron-folic acid supplementation and dietary diversification through timely initiation of antenatal care.

## 1. Introduction

A newborn's weight at birth is a vital indicator of maternal nutritional status and fetal health [1]. Globally, low birth weight (LBW) constitutes a major public health problem and is one of the main causes of newborn death [2, 3]. Every year, more than 20 million newborns are delivered with LBW [3, 4]; of these, over 96.5% occur in low-income countries. About 5.7 million African newborns are delivered with LBW [1, 5], and Ethiopia contributes 13% to this figure [6]. The magnitude of LBW varies across regions within countries [7–17].

With regard to its consequence, about 2.5 million and 23,091 newborns die every year due to LBW across the world and in Ethiopia, respectively [2, 18]. It also executes a remarkable burden on the social, economic, and healthcare system [7]. Low birth weight has various life-threatening and long-term consequences [1, 4, 19]. Newborns with birth weight less than 2500 g were about 20 times more likely to die than a newborn with normal birth weight [20]. Newborns with LBW stay in the hospital for a long time (average of 12.9 days) after birth which is far higher as compared to those with normal birth weight [21, 22]. This is based on epidemiological observations that newborns delivered with birth weight below 2500 grams are at higher risk of mortality when compared to those with normal birth weight [20].

Existing evidences from different parts of the world showed that the causes of low birth weight have been broadly grouped into individual-related, provider-related, and health service-related factors [16, 23–28].

The WHO has set a policy to reduce the prevalence of LBW by 30% at the end of 2025 [3]. This has been set for Ethiopia to a rate of 7% as the country is working in line with the global recommendations [29]. Promoting birth spacing, providing prenatal care, and strengthening the referral system and prevention of malaria and intestinal parasites, antibiotic treatment for bacterial infections, and prevention and control of hypertension during pregnancy were some of the interventions done to reduce LBW [3]. However, the progress in reducing global LBW has been stagnant since the year 2000 particularly during the most recent period from 2010 to 2015 [1]. Hardly was it rising in Ethiopia: 11% in 2011 and 13% in 2016 [6]. A recent systematic review in Ethiopia has come up with a pooled prevalence of LBW of 17.3% even though the study considered data from surveys done years ago [30].

Despite several studies being conducted in a different part of Ethiopia, the majority were reported from small settings and urban populations, which may underestimate the effect of possible risk factors from the rural population. This study is relatively a comprehensive study which covers more than 30 districts to determine predictors of LBW among newborns in public hospitals of Sidama Zone, South Ethiopia, where recent studies investigating the problem were not common. Thus, the study would give an input to programmatic interventions and policy considerations in the region and by extension to Ethiopia.

## 2. Methods

### 2.1. Study Setting, Design, and Population

An institution-based case-control study was conducted in Sidama Zone public hospitals from March 1 to May 5, 2019. Sidama Zone is one of 18 zones of the Southern Nations, Nationalities, and Peoples' Region (SNNPR) of Ethiopia. It is located 272 km to the south east of Addis Ababa, the capital city of Ethiopia. The zone has a total area of 6981.8 square kilometers and is administratively divided into 31 districts and 6 town administrations. According to the 2018 estimate, the zone has a total population of 4,294,730 (49% females and 51% males), and the expected number of deliveries was 148,598. Currently, 15 hospitals, 126 health centers, 531 health posts, and 107 private and 7 NGO clinics are providing health service for the population. According to the zonal health department report, the proportion of women who delivered in the health facilities in Sidama Zone in the year 2018/2019 was 71.6%. All mothers with their newborns delivered at public hospitals in Sidama Zone were considered source populations for cases and controls. Cases were newborns with birth weight < 2500 g, and controls were newborns with birth weight ≥ 2500 g.

### 2.2. Sample Size Determination

Sample size was estimated using OpenEpi software with the consideration of the proportion of rural women among controls (24.8%) and the odds ratio of 2.1 [27] and with the assumptions of the confidence level of 95%, power of 80%, and case to control ratio of 1 : 2. After additional consideration of the 10% nonresponse rate, the total sample size was 354 (118 cases and 236 controls).

### 2.3. Sampling Procedure

Five hospitals were selected out of 15 public hospitals by using the simple random sampling lottery method. The required sample was allocated proportionally to each hospital based on the number of LBW deliveries reported from each hospital in the year preceding the survey. Cases were selected consecutively, and two controls were also included consecutively for each case. Women with twin deliveries and who were seriously ill during the data collection were excluded from the study.

### 2.4. Data Collection Procedure and Tools

Data were collected by 10 female diploma holder midwives who speak the local languages fluently. Five male midwives with a bachelor degree were recruited as supervisors. Data collectors interviewed mothers using an interviewer-administered structured questionnaire. The serum hemoglobin level of mothers was extracted from their medical records since the hemoglobin level test is routinely done for all mothers who come for delivery services. The questionnaire was adapted by reviewing different related literatures [7, 22, 23, 29–41].

### 2.5. Measurements

Weights of the newborns were measured by using a digital Seca balance scale (Germany) to the nearest 1 g. The scale was adjusted to the zero level before weighing each newborn. Height of the mothers was measured using a height board while the mother was in standing position. Each height was taken to the nearest 0.01 m. MUAC of the mother was measured with a nonstretchable standard tape to the nearest 0.1 cm. Minimum Dietary Diversity for Women (MDD-W) was measured by the ten questions developed by FAO and FANTA as a proxy indicator to reflect the micronutrient adequacy of women's diets. Those women who replied “yes” for each food item interviewed were taken as “consumed,” and those who replied “no” did not consume anything in the previous 24 hours [31].

### 2.6. Data Quality Management

Detailed training was given to both data collectors and supervisors in the context of the study and measurement of weight of the newborns and height and MUAC of the mothers. The questionnaire was prepared in English and translated to Amharic then retranslated back to English. A pretest was conducted on 5% of the total sample size one week before the actual data collection. The data collection was supervised, and the collected data were checked for completeness and consistency on a daily basis.

### 2.7. Data Analysis

The collected data was checked for completeness and then coded, checked, and entered into EpiData version 4.2.1. After completing the entry in EpiData software, the data was exported to SPSS version 20 for cleaning and analysis. Data transformation was done where appropriate. Crosstabulations were made to check for the fulfillment of chi-squared assumptions. Descriptive analysis was conducted and presented using frequency tables and summarized using the mean and standard deviation. To determine the wealth index of participants, a principal component analysis was carried out after checking the fulfillment of assumptions. Women's household wealth was ranked into five groups (quintiles) as poor, second, medium, fourth, and highest from the lowest to the highest quintiles. Bivariate logistic regression was carried out, and variables with *p* value ≤ 0.25 were entered into multivariable logistic regression. The multivariable logistic regression model was built using the backward likelihood ratio method in the final model. Model fitness was assessed by using the Hosmer and Lemeshow goodness-of-fit test. Finally, the determinants of LBW were reported by an adjusted odds ratio (AOR) with 95% confidence interval (CI).

## 3. Results

### 3.1. Sociodemographic Characteristics of the Study Participants

One hundred eighteen cases (birth weight < 2500) and 236 controls (birth weight ≥ 2500) participated in the study. The mean birth weight was 2089 g (SD ± 260 g) and 3181 g (SD ± 358 g) for the cases and controls, respectively. More than half of the cases (61 (51.7%)) and controls (140 (59.3%)) were males. Majority of mothers of the cases (107 (90.7%)) and controls (215 (91.1%)) were married; most of the mothers of the study participants among cases (81 (68.6%)) and controls (176 (74.6%)) were between the age of 20 and 34 years. Similarly, 107 (90.7%) mothers of cases and 148 (62.7%) mothers of controls were taking inadequate minimum dietary diversity (Table 1).

### 3.2. Obstetric and Other Health-Related Characteristics of Mothers of the Study Participants

Fifty-one (43.2%) mothers of cases and 82 (34.7%) mothers of controls were Prim-Para. Similarly, 23 (34.3%) mothers of cases and 44 (28.6%) mothers of controls had a history of low birth weight, and also 18 (26.9%) mothers of cases and 31 (20.1%) controls had a history of stillbirth. With regard to their pregnancy intention, 50 (42.4%) mothers of cases and 98 (41.5%) mothers of controls had unintended pregnancy. Thirty-four (28.8%) mothers of cases and 54 (22.9%) mothers of controls were having preterm deliveries (Table 2).

### 3.3. Factors Associated with Low Birth Weight

A total of thirteen variables with *p* value < 0.25 in the bivariate analysis were included in multivariable logistic regression. The odds of being rural dweller women was 3.51 times higher among cases (low birth weight babies) than among controls (normal birth weight babies) as compared to being urban dweller women (AOR = 3.51, 95% CI (1.91-6.45)). The likelihood of initiating antenatal care late was 3.22 times more among cases than among controls when compared with timely initiation of antenatal care (AOR = 3.22, 95% CI (1.47-7.14)). The probability of having pregnancy-induced hypertension was 4.49 times higher among mothers of the cases than among mothers of the controls as compared to not having pregnancy-induced hypertension (AOR = 4.49, 95% CI (1.94-10.38)). The odds of not taking iron and folic acid during pregnancy was 3.92 times higher among mothers of the cases than among mothers of the controls when compared with taking iron and folic acid (AOR = 3.92, 95% CI (1.80-8.50)). The likelihood of having Mid-Upper Arm Circumference (MUAC) < 23 cm was 4.27 times higher among mothers of the cases than among mothers of the controls as compared to having MUAC ≥ 23 cm (AOR = 4.27, 95% CI (2.24-8.12)). The probability of having inadequate dietary diversity was 3.75 times higher among cases than among controls as compared to having adequate dietary diversity (AOR = 3.75, 95% CI (1.64-8.57)) (Table 3).

## 4. Discussion

The odds of being rural dweller women was higher among cases (low birth weight babies) than among controls (normal birth weight babies) as compared to being urban dweller women. The low accessibility of health facility and maternal health service utilization in a rural area might increase the likelihood of having LBW infants. This finding was consistent with studies conducted in Ethiopia and Zimbabwe [27, 32, 33]. But it is inconsistent with a study conducted in another part of Ethiopia [13]. This discrepancy might be due to differences in the study settings where our study area covered both urban and rural hospitals and the latter study was conducted in an urban setting where a teaching and referral hospital was also included.

The likelihood of initiating antenatal care late was more among cases than among controls when compared with timely initiation of antenatal care. This finding is in line with studies conducted in Algeria, Nepal, and Ethiopia [26, 34–36]. This might be due to the fact that a woman who visits the health facility timely for ANC might have a better opportunity for dietary counseling and iron and folic acid supplementation, adequate time for iron absorption from the gastrointestinal tract, and timely detection and treatment of different infections [20]. As a result, the likelihood of having a low birth weight baby would be less as compared to their counterparts.

This study indicated that the maternal history of pregnancy-induced hypertension was found to be an independent factor contributing to low birth weight where the probability of having pregnancy-induced hypertension was higher among mothers of the cases than among mothers of the controls as compared to not having pregnancy-induced hypertension. This finding is in line with studies conducted in Ethiopia, Malaysia, Nepal, and Gambia [25, 26, 28, 37, 38]. This might be because blood vessel constriction reduces oxygen and nutrient perfusion to the placenta which leads to an increased risk of intrauterine growth restriction which leads to undernutrition which in turn exposes babies to low birth weight [3, 20].

The odds of not taking iron and folic acid during pregnancy was higher among mothers of the cases than among mothers of the controls when compared with taking iron and folic acid. This finding is comparable with studies conducted in different parts of Ethiopia [15, 16, 23, 24, 32, 35]. This might be because iron and folic acid intake during pregnancy reduces the risk of anemia and subsequently decreases the likelihood of having low birth weight newborns [24].

The likelihood of having Mid-Upper Arm Circumference (MUAC) < 23 cm was higher among mothers of the cases than among mothers of the controls as compared to having MUAC ≥ 23 cm. This might be explained by the mistaken perception of women that frequent and much diet consumption during pregnancy could lead to excessive fetal growth which they perceive would be beyond tolerance of the birth canal and pose difficulty during childbirth [32, 39]. Due to this, pregnant women might not take adequate food; thus, they might be prone to undernutrition and can have low birth weight newborns. The effect of malnutrition has an intergenerational continuum; if a mother was malnourished during pregnancy, the likelihood of having a low birth weight infant will be high. This finding was consistent with studies conducted in Ethiopia [23, 24, 40]. In this study, dietary diversity during pregnancy was found to be an independent contributing factor of low birth weight where the probability of having inadequate dietary diversity was higher among cases than among controls as compared to having adequate dietary diversity. The possible explanation for this might be the fact that an inadequately diversified diet lacks some essential nutrients which would be essential for fetal growth and development that consequently leads to low birth weight. This finding is in agreement with a study conducted in Ethiopia [24].

Even though the findings on the factors associated with LBW in this study were consistent with the majority of pocket studies in the country, it was not supported by a recent systematic review in Ethiopia [30]. This might be explained by the fact that two-thirds of the studies considered in the review were cross-sectional studies.

The present study has few limitations to consider. The self-reported nature of the determinants based only on the mothers' memory might have a recall bias, and there may also be an intra- or interobserver bias that might have led to misclassification of cases and controls. The data was collected from mothers who gave birth at the health facility limiting its generalizability to all women in the zone as those who gave birth at home have been left out.

## 5. Conclusion

The finding of this study showed that time of antenatal care initiation, the residence of participants, maternal malnutrition measured by Mid-Upper Arm Circumference and inadequate dietary diversity during pregnancy, not getting iron and folic acid supplementation, and having pregnancy-induced hypertension were found to be independent determinants of birth weight among newborn babies. So concerned health authorities and health professionals should strengthen the existing promotion on timely initiation of antenatal care and stick to iron and folic acid supplementation recommendations during the antenatal care. Nutrition education and capacity building should be strengthened during the prenatal care in the local setup and by extension to the country as a whole.

## Figures and Tables

**Table 1:** Sociodemographic characteristics of the study participants from public health hospitals of Sidama Zone, South Ethiopia, 2019.

Variables	Cases No. (%)	Controls No. (%)	*p* value
Sex of the newborn			
Male	61 (51.7)	140 (59.3)	0.173
Female	57 (48.3)	96 (40.7)	
Maternal age			
<20 years	21 (17.8)	33 (14.0)	
20 to 34 years	81 (68.6)	176 (74.6)	0.491
≥35 years	16 (13.6)	27 (11.4)	
Residence			
Urban	38 (32.2)	139 (58.9)	0.001
Rural	80 (67.8)	97 (41.1)	
Marital status			
Married	107 (90.7)	215 (91.1)	0.899
Separated and divorced	11 (9.3)	21 (8.9)	
Maternal level of education			
Unable to read and write	57 (48.3)	75 (31.8)	
Primary school	31 (26.3)	74 (31.4)	0.003
Secondary school	22 (18.6)	50 (21.2)	
Above secondary	8 (6.8)	37 (15.7)	
Occupation of mother			
Housewife	47 (39.8)	81 (34.3)	
Merchant	21 (17.8)	50 (21.2)	
Farmer	16 (13.6)	41 (17.4)	0.624
Student	20 (16.9)	32 (13.6)	
Employed	14 (11.9)	32 (13.6)	
Wealth index of household			
Lowest	31 (26.3)	58 (24.6)	
Second	13 (11.0)	39 (16.5)	0.453
Middle	21 (17.8)	46 (19.5)	
Fourth	34 (28.8)	51 (21.6)	
Highest	19 (16.1)	42 (17.8)	
MUAC			
<23 cm	58 (49.2)	38 (16.1)	0.001
≥23 cm	60 (50.8)	198 (83.9)	
Height			
<150 cm	15 (12.7)	13 (5.5)	0.018
≥150 cm	103 (87.3)	223 (94.5)	
Minimum dietary diversity			
Inadequate	107 (90.7)	148 (62.7)	0.001
Adequate	11 (9.3)	88 (37.3)	

**Table 2:** Obstetric-related characteristics of the study participants from public health hospitals of Sidama Zone, South Ethiopia, 2019.

Variables	Cases No. (%)	Controls No. (%)	*p* value
Parity			
Prim-Para	51 (43.2)	82 (34.7)	0.120
Multi-Para	67 (56.8)	154 (65.3)	
History of LBW			
Yes	23 (34.3)	44 (28.6)	0.392
No	44 (65.7)	110 (71.4)	
History of stillbirth			
Yes	18 (26.9)	31 (20.1)	0.267
No	49 (73.1)	123 (79.9)	
Birth interval			
≥2 years	47 (70.1)	115 (74.7)	0.484
<2 years	20 (29.9)	39 (25.3)	
History of abortion			
Yes	19 (16.1)	31 (13.1)	0.450
No	99 (83.9)	205 (86.9)	
Pregnancy status			
Intended	68 (57.6)	138 (58.5)	0.881
Unintended	50 (42.4)	98 (41.5)	
Anemia			
Yes	28 (23.7)	33 (14.0)	0.022
No	90 (76.3)	203 (86.0)	
Gestational age			
Preterm	34 (28.8)	54 (22.9)	0.223
Term	84 (71.2)	182 (77.1)	
Ever attended ANC			
Yes	105 (89.0)	216 (91.5)	0.438
No	13 (11.0)	20 (8.5)	
Time of ANC initiation			
1^st^ trimester	16 (15.2)	78 (36.1)	
2^nd^ trimester	29 (27.6)	71 (32.9)	0.001
3^rd^ trimester	60 (57.2)	67 (31.0)	
Number of ANC visit			
<4 times	70 (66.7)	130 (60.2)	0.26
≥4 times	35 (33.3)	86 (39.8)	
Dietary counseling			
Yes	52 (49.5)	120 (56.1)	0.27
No	53 (50.5)	94 (43.9)	
Iron and folic acid supplementation			
Yes	15 (14.3)	104 (48.1)	0.001
No	53 (85.7)	112 (51.9)	
Pregnancy-induced hypertension			
Yes	28 (23.7)	29 (12.3)	0.005
No	90 (76.3)	207 (87.7)	

**Table 3:** Determinants of LBW among newborns delivered at public hospitals of Sidama Zone, South Ethiopia, 2019.

Variables	Cases No. (%)	Controls No. (%)	COR (95% CI)	AOR (95% CI)
Residence				
Rural	80 (67.8)	97 (41.1)	3.03 (1.90-4.80)	3.51 (1.91-6.45)
Urban	38 (32.2)	139 (58.9)	1	1
Time of ANC initiation				
1^st^ trimester	16 (15.2)	78 (36.1)	1	1
2^nd^ trimester	29 (27.6)	71 (32.9)	2.17 (1.33-3.84)	0.80 (0.39-1.66)
3^rd^ trimester	67 (57.2)	67 (31.0)	4.35 (2.27-8.33)	3.23 (1.47-7.14)
Pregnancy-induced HTN				
Yes	28 (23.7)	29 (12.3)	2.22 (1.25-3.95)	4.49 (1.94-10.38)
No	90 (76.3)	207 (87.7)	1	1
Iron & folic acid supplementation				
Yes	15 (14.3)	104 (48.1)	1	1
No	90 (85.7)	112 (51.9)	5.57 (3.03-10.2)	3.92 (1.80-8.5)
Mid-Upper Arm Circumference				
<23 cm	58 (49.2)	38 (16.1)	5.04 (3.05-8.3)	4.27 (2.24-8.1)
≥23 cm	60 (50.8)	198 (83.9)	1	1
Dietary diversity during pregnancy				
Inadequate	107 (90.7)	148 (62.7)	5.78 (2.95-11.3)	3.75 (1.64-8.5)
Adequate	11 (9.3)	88 (37.3)	1	1

## Data Availability

The [SPSS/EXCEL] data used to support the findings of this study are available from the corresponding author upon request.

## Abbreviations

ANC: Antenatal care
AOR: Adjusted odds ratio
COR: Crude odds ratio
FANTA: Food and Nutrition Technical Assistance
FAO: Food and Agriculture Organization
LBW: Low birth weight
MDD-W: Minimum Dietary Diversity for Women
MUAC: Mid-Upper Arm Circumference.
